# Transcriptome Insights into Candidate Genes of the *SWEET* Family and Carotenoid Biosynthesis during Fruit Growth and Development in *Prunus salicina* ‘Huangguan’

**DOI:** 10.3390/plants12193513

**Published:** 2023-10-09

**Authors:** Zhimin Lin, Xiaoyan Yi, Muhammad Moaaz Ali, Lijuan Zhang, Shaojuan Wang, Faxing Chen

**Affiliations:** 1 Fujian Academy of Agricultural Sciences Biotechnology Institute, Fuzhou 350003, China; 2 College of Horticulture, Fujian Agriculture and Forestry University, Fuzhou 350002, China; 1210305019@fafu.edu.cn (X.Y.); muhammadmoaazali@yahoo.com (M.M.A.); 1210305020@fafu.edu.cn (L.Z.); 3210330057@fafu.edu.cn (S.W.)

**Keywords:** *Prunus salicina*, sugar metabolism, carotenoid biosynthesis, fruit development, transcriptome

## Abstract

The Chinese plum (*Prunus salicina* L.) is a fruit tree belonging to the Rosaceae family, native to south-eastern China and widely cultivated throughout the world. Fruit sugar metabolism and color change is an important physiological behavior that directly determines flavor and aroma. Our study analyzed six stages of fruit growth and development using RNA-seq, yielding a total of 14,973 DEGs, and further evaluation of key DEGs revealed a focus on sugar metabolism, flavonoid biosynthesis, carotenoid biosynthesis, and photosynthesis. Using GO and KEGG to enrich differential genes in the pathway, we selected 107 differential genes and obtained 49 significant differential genes related to glucose metabolism. The results of the correlation analyses indicated that two genes of the SWEET family, evm.TU.Chr1.3663 (*PsSWEET9*) and evm.TU.Chr4.676 (*PsSWEET2*), could be closely related to the composition of soluble sugars, which was also confirmed in the ethylene treatment experiments. In addition, analysis of the TOP 20 pathways between different growth stages and the green stage, as well as transient overexpression in chili, suggested that capsanthin/capsorubin synthase (PsCCS) of the carotenoid biosynthetic pathway contributed to the color change of plum fruit. These findings provide an insight into the molecular mechanisms involved in the ripening and color change of plum fruit.

## 1. Introduction

Plum (*Prunus salicina* L.), belonging to the Rosaceae family within the genus Prunus, is a significant stone fruit valued by consumers for its distinctive flavor and economic importance [1]. Plums have been shown to improve digestion, lower blood pressure, protect the liver, and reduce colon cancer due to the anthocyanins and flavonoids found in the fruit. *Prunus salicina* ‘Huangguan’ is the local characteristic variety of Fujian, and it is a yellow-skinned, yellow-fleshed, early-ripening variety of plum derived from Plunus, which is used for fresh consumption and is characterized by its bright color, juiciness, and sweet-sour flavor [2].

Fruit development is a complex process that can be divided into two main periods of rapid cell division following pollination, fertilization, and subsequent cell growth [3]. A number of physiological and biochemical changes normally occur during fruit development, including fruit growth and changes in flavor, nutrients, color, and texture [4]. Fruit is an important fresh plant product for the human diet because of its relatively high sugar content [5]. The total amount of soluble sugars in the fruit usually varies as the fruit grows, peaking at maturity or ripening [6,7]. In plums, glucose is the most abundant sugar in the early stages of development, while sucrose increases during ripening [8]. Sorbitol is the main product of photosynthesis in the Rosaceae family. Its accumulation in plums is paralleled by increased sucrose catabolism in climacteric fruit [9]. Furthermore, the sugar metabolism in leaves and fruits of non-climacteric plums has been reprogrammed towards increased sorbitol synthesis, as shown by studies on two Japanese plum (*Prunus salicina* Lindl.) cultivars [10]. Sugar content also affects plum quality, except for total phenolics and anthocyanins, as shown by the analysis of 178 plum varieties [11]. Indeed, sucrose in fruit can be converted to fructose and glucose by neutral converting enzymes. Glucose and fructose can be phosphorylated to glucose-6-phosphate (G6P) and fructose-6-phosphate (F6P) by hexokinase (HK) and fructokinase (FK), respectively [12].

In plants, the process of soluble sugar synthesis is mainly mediated by the activity of various enzymes or transcriptional levels of genes involved in sugar metabolism and transport [13]. Typically, the breakdown of sucrose to glucose, fructose, or UDP-glucose is mediated by three families of enzymes: invertases (INVs), sucrose synthases (SuSys), and sucrose phosphate synthases (SPSs) [14]. Sugar will eventually be exported as a transporter (SWEET), which is a novel sugar mono-transporter protein that is a bidirectional sugar transporter and facilitates the diffusion of sugars along a concentration gradient [15,16]. It is also involved in important physiological processes of plant growth and development by regulating the transport, distribution, and storage of carbohydrates in plants [17]. In tomatoes, the SWEETs are highly expressed in the developing fruit, and transporter *SlSWEET15* mediates the efflux of sucrose from the released phloem cells into the plasma membrane of the fruit parenchyma during fruit development [18]. Furthermore, SWEETs are not only involved in sugar transport and affects sugar levels but also regulate important physiological processes related to abiotic stress, growth, and fertility in plants by regulating sugar transport [19].

Carotenoids are a group of terpenoids made up of 40 carbon skeletons that are derived from the MEP pathway [20]. Plums are rich in carotenoids [21]. Analysis of transcriptomic and metabolomic data from plum suggests that carotenes and carotenoids may be related to the color of Li fruit but do not play a dominant role [22]. In chili, strong induction of *CaCCS1* causes capsanthin to accumulate in mature fruits [23]. It was also shown that the regulation of *CCS* expression would affect the chili yellow phenotype [24]. 

While our previous transcriptomic research has indicated the significance of *bZIP* transcription factors in governing the accumulation of glucose and fructose [2], the precise biological mechanisms responsible for sugar accumulation and carotenoid biosynthesis during the ripening of *Prunus salicina* ‘Huangguan’ plums have yet to be fully understood. In this study, we collected fruit samples at six different developmental stages of *Prunus salicina* ‘Huangguan’ and conducted a comprehensive analysis of gene expression changes in both the sugar metabolism and carotenoid biosynthesis pathways. This analysis included genes such as *SUT*, *SWEET*, *SOT*, *HT*, *ERD61*, *CCS*, and more, using transcriptomics. To gain a comprehensive understanding of the relationship between genes and sugar content, we also examined changes in soluble sugars, including sucrose, fructose, and other major sugar components. Furthermore, to explore the impact of the *CCS* gene on fruit coloration in plum fruits, we conducted experiments involving the overexpression of *CCS* in chili peppers, shedding light on its influence on fruit color transformation.

## 2. Results

### 2.1. Determination of Soluble Sugar Content in Plum Fruit during the Developmental Period

**Figure 1:**
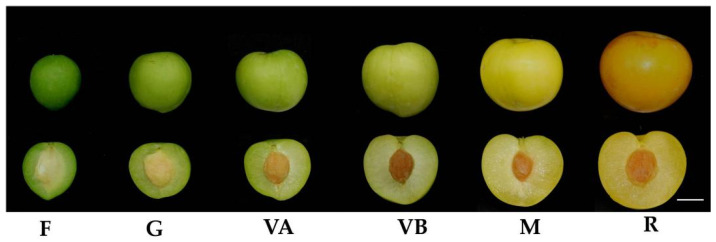
The ‘Huangguan’ plum at different fruit developmental stages. F—fruitlet, G—green, VA—veraison A, VB—veraison B, M—mature, and R—ripened. Scale = 1 cm.

In order to analyze the changes in sugar composition during the development of the plum fruit, fruit was taken from six different periods (Figure 1). The content of total soluble solids (TSS) and four soluble sugars in plum fruits at different developmental stages were determined by HPLC. The results showed a gradual increase from 5.93% TSS at the young fruit stage to 10.50% at the ripe stage (Figure S1A). The total soluble sugar content of 74.99 mg/g at maturity also showed a gradual increase (Figure S1B). The glucose content increased continuously throughout the development of the plum fruit, from a minimum of 14.71 mg/g at the young fruit stage to 42.30 mg/g at the ripe stage (Figure S1C). The glucose content increased continuously as the plum developed and ripened, from a minimum of 14.71 mg/g at the young fruit stage to 42.30 mg/g at the ripe stage. The fructose and sucrose contents followed the same trend, with the fructose and sucrose contents decreasing from 4.54 mg/g and 1.40 mg/g at the young fruit stage to 2.01 mg/g and 1.19 mg/g, respectively, at the green fruit stage, and then increased continuously from the green fruit stage to the ripe fruit stage (Figure S1D,E). Thus, soluble solids and major sugars generally showed an increasing trend with the developmental stage. 

### 2.2. Quality Assessment through Transcriptome Analysis

**Figure 2:**
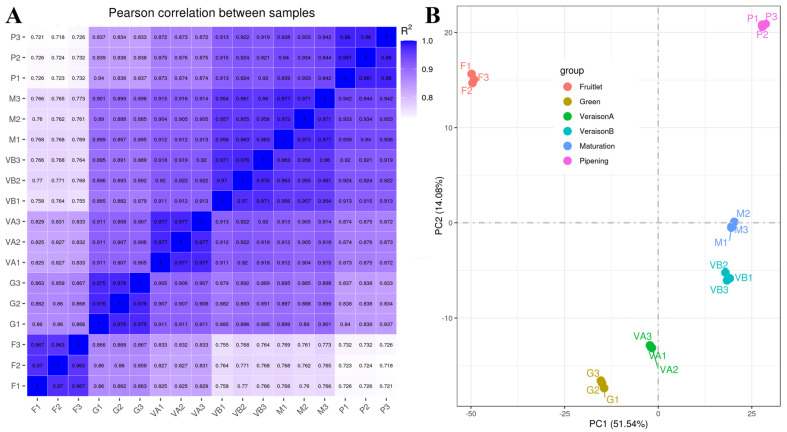
Gene expression-based Pearson (*n*) correlation (**A**) and principal component analysis (PCA) (**B**) among different developmental stages of plum. Coloring is used to distinguish between samples: red, brown, green, cyan, blue, and purple represent fruitlet, green, veraison A, veraison B, maturation, and ripening, respectively.

Transcriptome sequencing of six developmental stages of plum fruit yielded 123.28 Gb of raw reads, which were quality filtered to yield 121.57 Gb of clean bases, with an average mapping to the reference genome of 85.77%, and average Q20 (more than 96% of bases with an error rate < 1%) and Q30 (more than 90% of bases with an error rate < 0.1%) quality values of 97.53% and 93.02%, respectively. The GC% content of each sample base was approximately 45%. Pearson (*n*) correlation analysis and principal component analysis (PCA) were performed on 18 samples to determine the gene expression level of each sample based on the FPKM value (Figure 2A,B). The correlation results showed that the intra-group correlation coefficients of the six stages’ samples were all above 0.95. The eighteen samples were divided into six groups in the PCA results and corresponded to the six periods of fruit development, indicating that the intra-group correlation of the samples was high and that there was good correlation and difference between the samples.

### 2.3. DEGs’ Analysis during Fruit Development

**Figure 3:**
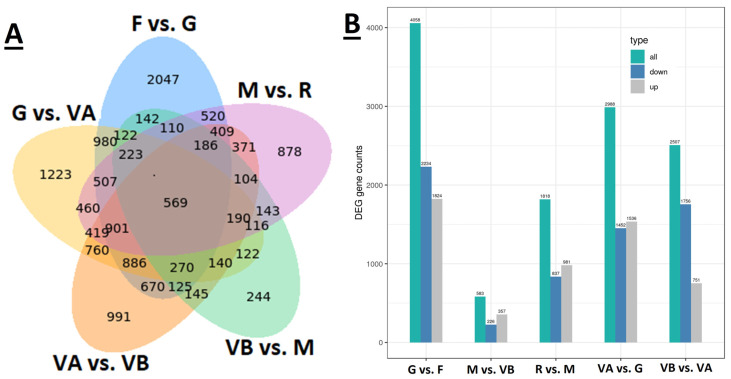
(**A**) Venn diagram of DEGs showing comparisons of samples collected at six different developmental stages of plum; (**B**) Numbers of up-regulated and down-regulated genes after comparisons of samples collected at six different developmental stages of plum. F—fruitlet, G—green, VA—veraison A, VB—veraison B, M—mature, and R—ripened.

The sample from different growth stages of plum were divided into five comparison groups, i.e., green vs. fruitlet, veraison A vs. green, veraison B vs. veraison A, maturation vs. veraison B, and ripening vs. maturation. The transcriptome data were screened according to the threshold conditions of FDR < 0.05 and |log2FC| > 1. We identified 14,973 DEGs associated with transitions to different developmental stages from F to R, of which 2047, 1223, 991, 244, and 878 DEGs were unique to F/G, G/VA, VA/VB, VB/M, and M/R, respectively (Figure 3A). A total of 11,954 differentially expressed genes were obtained, with the highest number of 4058 differentially expressed genes in the green vs. fruitlet group, which was much higher than the other comparison groups. The lowest number of DEGs was only 583 in the maturation vs. veraison B group, indicating that there were more DEGs in the early stage of fruit development than in the late stage. It was speculated that more complex physiological changes occurred in the early stage of fruit development. Among all the comparison combinations, the comparison between the green and fruitlet stages, as well as the comparison between veraison B and veraison A, exhibited a greater number of up-regulated genes compared to down-regulated genes. Notably, the green vs. fruitlet group displayed the highest count of down-regulated differentially expressed genes (DEGs) among all the comparison groups, with 1824 down-regulated genes, while the other developmental stage comparison groups had a higher count of up-regulated DEGs (Figure 3B). 

### 2.4. KEGG Pathway Enrichment Analysis of DEGs

The DEGs were characterized using KEGG pathway enrichment analysis to explore the relevant biological functions and to highlight functional differences between developmental stages. From the KEGG enrichment results, the pathways associated with sugar metabolism in the TOP20 are primarily sucrose and starch metabolism, glycolysis/glycolysis, and pentose/glucuronic acidic interconversion (Figure S2). DEGs analyses showed a significant enrichment in photosynthesis, carbon metabolism, starch metabolism, and sugar metabolism during pre-fruit development (Figure S2A,B). In addition, a high percentage of DEGs in the pathways related to sugar metabolism were up-regulated in the pre-fruit developmental period and down-regulated in the post-fruit developmental period (Figure S2C,D). They were also enriched in biological pathways such as phenylpropane biosynthesis, photosynthesis, hormone signaling, and amino acid metabolism pathways during fruit development.

### 2.5. Sugar Metabolism-Related DEGs

**Figure 4:**
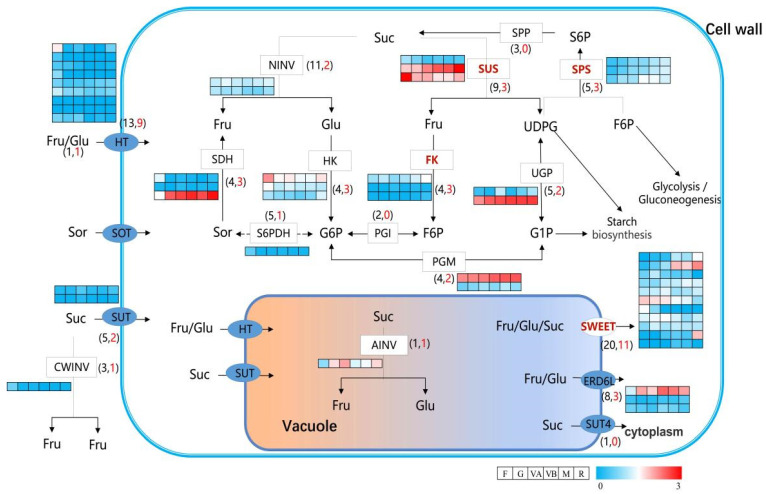
Expression of sugar metabolism-related DEGs at different fruit developmental stages of plum. A pathway of galactose metabolism and key enzymes involved are also shown. The key enzymes discussed in this study are highlighted in red. SPP, sucrose-phosphatase; NINV, neutral invertase; CWINV, cell wall invertase; SUS, Sucrose synthase; SPS, sucrose phosphate synthase; SDH, sorbitol dehydrogenase; HK, hexokinase; FK, fructokinase; UGP, UDPG-pyrophosphorylase; S6PDH, sorbitol-6-phosphate dehydrogenase; PGI, phosphoglucoisomerase; PGM, phosphoglucomutase; HT, hexose transporter; SOT, sorbitol transporter; SUT, sucrose transporter; AINV, acid invertase; SWEET, sugars will eventually be exported transporter; SUT4, sucrose transporter 4; ERD6L, early response to dehydration like 6. Black represents key genes, and red represents the number of significantly differentially expressed genes.

Based on the glucose metabolism-related genes annotated in the KEGG pathways, a total of 49 DEGs were obtained as candidate genes related to the glucose metabolism pathway (Figure 4). Different sucrose converting enzymes (INV) differed among the six stages of fruit development. Among them, CWINV had the highest expression at the fruitlet stage, NINV had the highest expression at the green stage, and AINV had the highest expression at the veraison A stage. Among the *SUS* genes related to sucrose metabolism, three *SUS* genes were differentially expressed, two of which had opposite trends in expression abundance during fruit development, and the three *SPPs* had higher expression at later stages of fruit development compared to earlier stages. Meanwhile, three *FKs*, three *HKs*, two *UGPs*, and two *PGMs* were shown to be differentially expressed during fruit development, with evm.TU.Chr1.2546 (*PGM*) and evm.TU.Chr3.153 (*UGP*) consistently up-regulated during fruit development. In addition, the expression of *SUT* was low at all six developmental stages, with most of the *HTs* having the highest expression at fruitlet stage and very low or no expression at the maturation and ripening stages. Interestingly, we revealed 11 differentially expressed *SWEET* genes in the pathway. Evm.TU.Chr1.3663 was consistently up-regulated during fruit development and evm.TU.Chr4.676 was consistently down-regulated during fruit development. Given the divergent expression trends observed in both *SWEET* genes, it is conceivable that their functional roles involve contrasting regulatory mechanisms—one being activated through up-regulation, while the other operates via down-regulation.

### 2.6. Structure and Motif Analysis of PsSWEET Genes and Correlation with Soluble Sugar Content

**Figure 5:**
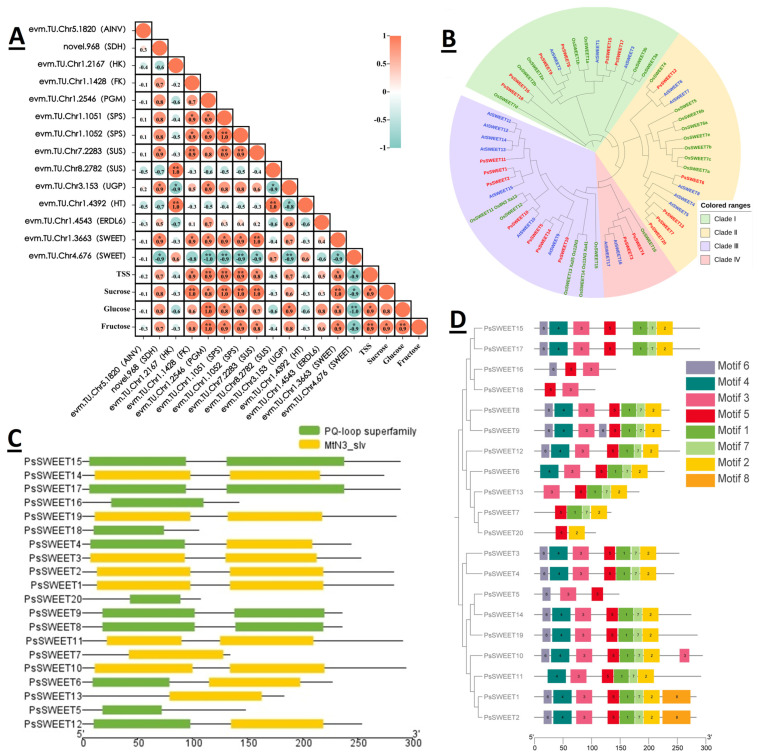
(**A**) Correlation analysis of sugar transporter genes with changes in sugar content. The symbols “*” and “**” are used to indicate significance levels corresponding to *p*-values of ≤0.05 and ≤0.01, respectively. (**B**) Cluster analysis of two *SWEET* family genes in plum with Arabidopsis and rice. Colors are used to distinguish between the species, i.e., red, blue, and green for plum, Arabidopsis, and rice, respectively. (**C**) *PeSWEET* gene structures; yellow color indicates the exons; green color shows the introns. (**D**) Conserved motifs analysis of *PsSWEET* genes.

To further validate the candidate genes related to sugar metabolism, 7 key DEGs were selected for qRT-PCR validation. The 14 DEGs were further screened for significance correlation with sugar metabolism, and correlation analyses were performed with TSS, sucrose, fructose, and glucose content. The results showed that evm.TU.Chr1.1428 (FK) was significantly and positively correlated with sucrose and TSS content, evm.TU.Chr1.2546 (PGM), evm.TU.Chr1.1051 (SPS), evm.TU.Chr1.1052 (SPS), and evm.TU. Chr7.2283 (SUS) were positively correlated with TSS; sucrose, fructose, and glucose content were positively correlated; and evm.TU.Chr3.153 (UGP) was significantly positively correlated with glucose content (Figure 5A). The results of gene clustering analysis showed that *PsSWEET9* and *AtSWEET2* had a high similarity of 72%; *PsSWEET2* and *AtSWEET15* had a high similarity of 99% (Figure 5B). There were 11 differentially expressed *PsSWEETs* in fruit, which were analyzed by correlation with TSS and the soluble sugar content in fruit. The evm.TU.Chr1.3663 (SWEET) was positively correlated with soluble sugar content and evm.TU.Chr4.676 (SWEET) was negatively correlated with soluble sugar. For full validation, we showed that evm.TU.Chr1.3663 (SWEET) was up-regulated and evm.TU.Chr4.676 (SWEET) was down-regulated compared to the control as the fruit ripened by RT-qPCR analysis of ethylene-mediated ripening of plum fruits (Figure S4). The expression patterns of these 7 DEGs obtained by RT-qPCR in six fruit development stages were basically consistent with the transcriptome results, demonstrating the accuracy and reliability of transcriptome sequencing (Figure S3A–G).

Analysis of the structure by conserved motifs, exons, and intron pairs of the genes helped to further understand the evolution of the *PsSWEETs* gene family members. Structural analysis of 20 *PsSWEET* genes showed that the two major structural domains, the PQ-loop superfamily and MtN3_slv, are highly similar (Figure 5C). The conserved motifs of *20 PsSWEET* genes were predicted, and the range of conserved motifs varied from 2 to 8 (Figure 5D). Most *PsSWEET* genes had more than 5 exons, while genes such as *PsSWEET15* and *PsSWEET2* had 8 exons, respectively. From an evolutionary point of view, the *PsSWEET* gene family had the same characteristics and was likely to have the same function.

### 2.7. Differentially Expressed Carotenoid Biosynthetic Genes 

**Figure 6:**
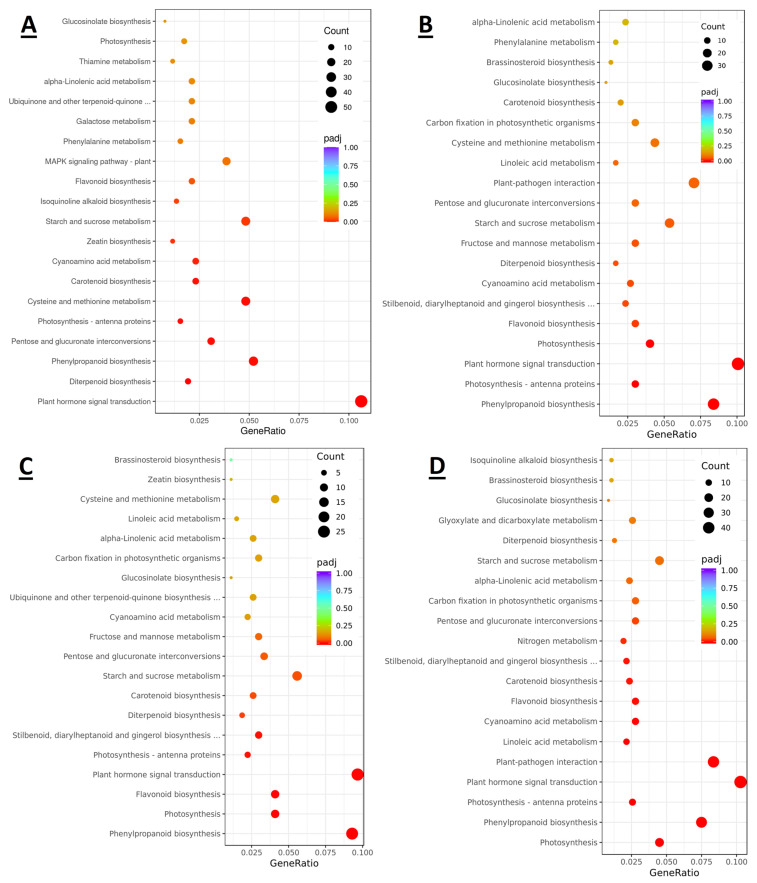
Results of the KEGG enrichment analysis of differentially expressed genes (DEGs) at different developmental stages of plum. (**A**) Scatterplot of enriched KEGG pathway between G and VA stage. (**B**) Scatterplot of enriched KEGG pathway between G and VB stage. (**C**) Scatterplot of enriched KEGG pathway between G and M stage. (**D**) Scatterplot of enriched KEGG pathway between G and P stage.

The TOP 20 enriched KEGG pathways of the DEGs for each period (Figure S2), compared with each other, indicated that the pathway of carotenoid biosynthesis was involved at the green stage (Figure 6). However, the carotenoid biosynthesis pathway of the TOP 20 was not enriched at the young fruit stage. Therefore, the color change period is an important physiological stage for carotenoid biosynthesis in fruit development. The data from the TOP 20 showed that there are 12 DEGs between the G and VA stages, 6 DEGs between the G and VB stages, 7 DEGs between the G and M stages, and 11 DEGs between the G and P stages (Figure S6). In this study, we focus on capsanthin/capsorubin synthase because it undergoes a significant up-regulation of the transcript levels compared to the green fruit phase (G), with the exception of the F stage (Figure S7). The results of the transient expression of *CCS* genes in chili showed that overexpression of CCS genes promoted earlier color change in chili fruits (Figure S8).

## 3. Discussion

### 3.1. Sugar Metabolism in Plum

Sugar is a basic component required for plant development, providing an energy source and carbon skeleton for plant growth and development [25]. In fruit, sugar is an important indicator of fruit flavor, and sugar accumulation during fruit ripening has always been a focus of fruit quality research [26]. In response to the complex trait of fruit sensory quality, transcriptome sequencing has been used to identify candidate genes related to sugar metabolism, providing a basis for understanding the molecular mechanisms that regulate sugar metabolism and accumulation [27,28].

Sugar transporter proteins play a key role in many fundamental processes of plant growth and development. Although no galactose transporter proteins have yet been identified in fruit species, about 60 Arabidopsis genes have been shown to encode putative monosaccharide transport proteins [29]. Most plants have a variety of sucrose transporter proteins (SUTs) with different functions for loading and unloading sucrose in different tissues [30]. Sugar transporter protein activity is regulated at several levels, including transcriptional and post-translational [31]. Several monosaccharide and sucrose transporter proteins have been identified in plant plasma or vesicle membranes, including sucrose transporter proteins (SUTs), and monosaccharide transporter proteins (MSTs) and sugar will eventually be exported transporter proteins (SWEETs) [32,33,34]. The SUT superfamily of sucrose transporters was originally described as plasma membrane resident proteins. However, studies have shown that SUTs are capable of chelating and transporting subcellular and intercellular cargo through intercellular connecting filaments [35]. Overexpression of *PbSUT2* in transgenic tomato resulted in an increase in the net photosynthetic rate of leaves and the sucrose content of ripe fruits, as well as a decrease in the glucose, fructose, and total soluble sugar content of ripe fruits [36]. Monosaccharide transport proteins (MSTs) play a key role in carbon transport and the efficient distribution of sugars, mediating the intercellular and long-distance distribution of a wide range of monosaccharides [37].

### 3.2. Role of SWEETs in Regulating Sugar Metabolism 

SWEET is widespread in plants, and members of the SWEET family have been isolated from rice, Arabidopsis, wheat, soybean, tomato, grape, loquat, etc., and different genes can transport different sugars [15,38,39,40,41,42,43]. In plants, SWEETs typically have seven transmembrane structural domains—single transporters that transport hexose and sucrose and mediate the efflux of sugars into the extracellular body [34,44,45]. In addition, SWEET has multiple physiological functions, including pollen feeding, nectar secretion, seed germination, phloem loading, and pathogen feeding [46]. In rice, 21 members of the SWEET family have been identified and play important roles in stress response [47]. *OsSWEET14* and *OsSWEET11* play an important role in rice grain filling by providing sucrose to the endosperm through sucrose efflux bodies [48]. *AtSWEET11* and *AtSWEET12* are highly expressed in Arabidopsis leaf tissue and are important sucrose export transporters [49], including *AtSWEET13* associated with pollen development [50]. Analysis of *MdSWEET* gene polymorphisms in 188 apple cultivars and the soluble sugar content of ripe fruit show that three genes were significantly associated with fruit sugar content in apples, including *MdSWEET2e*, *MdSWEET9b,* and *MdSWEET15a* [51]. *SISWEET7a* and *SISWEET14* in tomatoes transport fructose, glucose, and sucrose, and down-regulation of expression also resulted in taller and larger plants [52]. In our laboratory, previous transcriptomic analyses revealed the presence of differentially expressed SWEET genes related to sugar metabolism in plum fruit. However, the limited sample size prevented us from conducting a comprehensive analysis to determine whether these nine SWEET family genes were directly associated with sugar levels [2]. This study has initially identified a total of 20 SWEET genes within the sugar metabolism pathway in *Prunus salicina* ‘Huangguan’. Among these, 11 are directly linked to sugar transport (Figure 4). From the promoter cis-acting elements of the *PsSWEETs* genes, there are twenty-two major elements, including light responsive elements, cold stress, and ethylene responsive elements. Seven of the genes, including *PsSWEET9*, contain the ethylene responsive element (Figure S5A). The *PsSWEETs* promoter in Li plum mainly contains 15 transcription factor binding sites, including TCP, MYB, GRAS, ERF, BES1, NAC, and bZIP (Figure S5B). The results show that the expression levels of two genes, *PsSWEET9* and *PsSWEET2*, correlate with the soluble sugar content, including fructose, sucrose, and glucose, in plum. In contrast, *PsSWEET9* is positively correlated, whereas *PsSWEET2* is negatively correlated with soluble sugar contents (Figure 5A). Gene expression validation of *SWEETs* by the ethylene ripening process showed that the expression changes of *PsSWEET9* and *PsSWEET2* during fruit ripening are consistent in both transcriptome and RT-qPCR validation results (Figure S4). Therefore, the *PsSWEET9* and *PsSWEET2* genes are involved in the sugar metabolism pathway of plum.

### 3.3. Role of CCS in Accelerating Fruit Color Change

In Prunus fruits, carotenoid accumulation is determined by chromoplast development [53]. In plums, anthocyanins play a dominant role in color change, and carotenoids and carotenes are involved in color change [22]. Capsanthin-capsorubin synthase (CCS) is specifically expressed during fruit ripening [54] and regulates capsanthin synthesis and red color formation in chili fruit [55]. In the current study, we demonstrate a significant alteration in carotenoid biosynthesis, particularly during the green fruit stage (G). This change has been substantiated through KEGG enrichment analysis (TOP20) (Figure S2). The color change of the plum fruit, or G-stage, is, therefore, an important stage in the initiation of carotenoid biosynthesis. We detected notable alterations in the expression patterns of several carotenoid-related genes when compared to the G stage. These genes include *bHY*, *PSY*, *NCED2/NCED1*, *CCS*, and *PSY* (Figure S6). Quantitative analysis of *PsCCS* genes at six stages of plum fruit development showed that their expression levels were up-regulated with fruit development (Figure S7). The outcomes of transiently overexpressing the *PsCCS* gene in chili peppers demonstrate its role in accelerating the early onset of color change, as depicted in Figure S8. Therefore, the *CCS* gene may play a role in the color change of *Prunus salicina* ‘Huangguan’.

## 4. Materials and Methods

### 4.1. Plant Material and Fruit Sampling

*Prunus salicina* plums were grown in the perennial form within Gutian County, Ningde City, located in Fujian Province, China. Sampling occurred at intervals of 17, 34, 46, 59, and 74 days after flowering. At each of these time points, fruits were collected, encompassing six distinct stages of plum development. These stages were categorized as follows: the fruitlet stage (F), green stage (G), the stage when fruits began pre-coloring (VA), the late stage of fruit color transformation (VB), maturity stage (commercial maturity) (M), and the ripeness stage (physiological ripeness) (R). The classification was based on criteria such as the fruit shape index and skin color, as depicted in Figure 1.

For each of the five stages, fruits were collected, creating a mixed sample, and this process was repeated to form three replicate samples. Following the sampling, the fruit was cut into small pieces, promptly placed in liquid nitrogen, and preserved at a temperature of −80 °C until needed for further analysis. Each sample was divided into two portions: one intended for transcriptomics sequencing, and the other allocated for assessing total solids, soluble sugar content, and qRT-PCR analysis.

### 4.2. Determination of Sugar Content

Fruit soluble solids were determined using a hand-held digital refractometer, with three biological replicates for each sample. Fruit sucrose, glucose, fructose, and sorbitol contents were determined by high-performance liquid chromatography (HPLC), according to a previous method [56]. The sample injection volume and flow rate of the mobile phase (water/acetonitrile) was 10 μL and 1 mL·min^−1^, respectively. The Agilent XBridge BEH AMIDE had an inner diameter of 4.6 × 250 mm and a particle size of 5 μm. The column temperature was maintained at 35 °C. Sucrose, fructose, glucose, and sorbitol were detected at 210 nm. The content of the four soluble sugars was determined by an external standard method using retention time qualitatively and peak area quantitatively, with three independent biological replicates for each sample, and the sugar content was expressed in mg/g.

### 4.3. RNA Extraction and RNA-seq Library Construction and Sequencing

RNA extraction was carried out using an RNA extraction kit (Tiangen, code: DP441), the quality of RNA was detected by 1.2% agarose, and the concentration of RNA was determined and the purity of RNA was analyzed using Ultra trace bio-detector (nanodrop one, Thermo, Waltham, MA, USA). It accurately detects the integrity of RNA using the Agilent 2100 (Agilent Technologies, Santa Clara, CA, USA). The first step is to build and check the library. Eukaryotic mRNA was enriched on magnetic beads using Oligo (dT), and a fragmentation buffer was added to break the mRNA into short fragments. AMPure XP beads were then used to purify double-stranded cDNA. The purified double-stranded cDNA was first end-repaired, A-tailed, and attached to sequencing connectors, and then fragment size selection was performed using AMPure XP beads. Finally, PCR was performed, and the PCR products were purified using AMPure XP beads to obtain the final library. RNA-seq was performed on Illumina’s NovaSeq 6000 high-throughput sequencing platform, performed by Novogene Technologies Co., Ltd. (Beijing, China).

### 4.4. Transcriptome Analysis

TopHat v2.0.12 was used to compare clean reads from the *Prunus salicina* (GeneBanK: GCA_020226455.1) as the reference genome. Read counts in features were performed using HTSeq v0.6.1. Gene lengths and read counts that were mapped to genes were used to calculate FPKM values. Read counts for features were performed using HTSeq v0.6.1 25. To calculate FPKM values, gene lengths and read counts mapped to genes were used.

The differentially expressed genes (DEGs) between different growth stages were identified using the DESeq R package, and Benjamini-Hochberg adjusted *p* values < 0.05 were considered statistically significant. 

### 4.5. Transcriptome Data-Based Identification of SWEET Genes in Plum

The 17 *AtSWEETs* and 21 *OsSWEETs* protein sequences were obtained by downloading from the Arabidopsis Genome Database website (TAIR, http://arabidopsis.org/, accessed on 22 January 2023) and the Rice Genome Database website (RGAP, http://rice.plantbiology.msu.edu, accessed on 22 January 2023), respectively. The Hidden Markov Model (HMM) file for the conserved structural domain sequences of SWEET proteins in the Pfam database, identified by Pfam ID PF03083, was utilized and subsequently downloaded. Further, the simple HMM Search function in the TBtools software (https://github.com/CJ-Chen/TBtools/releases accessed on 5 October 2023) was used to filter out the candidate SWEETs with E-value < 1 × 10^−10^. The transcriptome data was used to construct a local Blast database for plum, and the protein sequences of SWEETs from *Arabidopsis thaliana* and rice were used for Blast comparison to obtain candidate genes. The candidate genes obtained in the above two ways were combined, the redundant sequences were removed, and then the genes that did not contain the conserved structural domains of the SWEET family were removed, resulting in 20 candidate genes for *PsSWEETs*.

### 4.6. Physico-Chemical Characterization of PsSWEETs

The online bioinformatics analysis website ProtParam tool (https://web.expasy.org/pr-otparam/, accessed on 27 January 2023), TMHMM Server v. 2.0 (http://www.cbs.dtu.dk/services/TMHMM/, accessed on 27 January 2023), SignalP-5.0 (http://www.cbs.dtu.dk/services/SignalP/, accessed on 27 January 2023) and Cell-PLoc 2.0 (http://www.csbio.sjtu.edu.cn/bioinf/Cell-PLoc-2/, accessed on 27 January 2023) were used to analyze the physicochemical properties, transmembrane structures, and signal peptides of PsSWEETs (Table S2). 

### 4.7. Phylogenetic Analysis of Plum, Arabidopsis, and Rice SWEETs

The sequences of *PsSWEETs*, *AtSWEETs*, and *OsSWEETs* were subjected to multiple sequence alignment using the ClustalW method in MEGA 7.0 software, and the phylogenetic tree was constructed using the maximum likelihood method for the SWEET family members of the three species, with the bootstrap value set at 1000, and the iTOL online website (https://itol.embl.de/, accessed on 28 January 2023) was used to modify and beautify the evolutionary tree.

### 4.8. Conserved Structural Domains and Conserved Motifs in PsSWEETs

Conserved structural domains were analyzed for PsSWEETs using the NCBI-CDD database (https://www.ncbi.nlm.nih.gov/cdd, accessed on 27 January 2023) and visualized using TBtools software. Conserved motifs of PsSWEETs were analyzed using the MEME (https://meme-suite.org/meme/tools/meme, accessed on 27 January 2023) website, the motif value was set to 8, other parameters were set as default values, and visualized using TBtools software.

### 4.9. Prediction of Promoter Cis-Acting Elements and Transcription Factor Binding Sites for PsSWEETs

The transcriptome reference genome (https://www.rosaceae.org/Analysis/9019655, accessed on 28 January 2023) was used to obtain the upstream sequences (1000 bp) of the *PsSWEEETs* genes, the PlantCARE website (http://bionformatics.psb.ugent.be/webtools/plantcare/html, accessed on 28 January 2023) and PlantTFDBE website (http://bionform-atics.psb.ugent.be/webtools/plantcare/html, accessed on 28 January 2023) were used to predict promoter cis-acting elements and transcription factors, and then TBtools software was used to visualize.

### 4.10. Ethylene Treatment

The control and treated groups were soaked in 600 mg/L ethylene solution and water, respectively, and the plum fruits were removed and dried after 30 min, placed in transparent perforated plastic boxes, and stored at room temperature for 3 days.

### 4.11. Gene Amplification and Transient Expression Vector Construction

Total RNA from plum fruit was reverse transcribed into cDNA as a template, and primers (Table S1) were designed to amplify the full length of evm.TU.Chr6.1365 (CCS), which has 1503 bp, using the primer 6.0. PCR system: 5XSF buffer 10 μL, 10 mM dNTP Mix 1 μL, 500 ng/μL DNA template 1 μL, 10 uM prime1 2 μL, 10 uM primer2 2μL, Phanta HS Super-Fidelity DNA Polymerase 1 μL, and adding ddH_2_O to 50 μL. The PCR protocol was as follows: Pre-denaturation 95 °C 1 min, denaturation 95 °C 10 s, annealing 60 °C 15 s, extension 72 °C 2 min, 35 cycles, with re-extension 72 °C 5 min. After running the gel by electrophoresis, the amplification products were recovered using the kit (TIANGEN, Beijing, China, code:DP204). The pCAMBIA1300BG plasmid was double digested with *Xba*I and *Sac*I, and the final vector p1300B-CCS was constructed using a one-step cloning method (Vazyme, Biotechnology, Nanjing, China, code:C113). The plasmid was introduced into *Agrobacterium tumefaciens* strains G3101 and stored at −80 °C.

### 4.12. Transient Expression on Peppers

Single colonies were picked and incubated overnight in YEB medium containing 50 mg/L Kanamycin and 100 mg/L rifampicin in a shaker at 28 °C. Centrifugation was performed at 5000 rpm at low speed, and the precipitate was dissolved in suspension, diluted to an OD600 of 0.8–1.0, and allowed to stand for 2–3 h at room temperature. Further, the bacterial cell suspension was injected into the cyan fruit of the pepper, and phenotypic changes were observed after three days.

### 4.13. RT-qPCR Analysis

The RNA was extracted from samples of six developmental stages of plum using the Tiangen kit and was reverse transcribed to cDNA using the Vazyme Reverse Transcription Kit (Vazyme, Nanjing, China, code: R212). The RT-qPCR analysis was performed on an ABI QuantStudio 1 Real-Time PCR System (Applied Biosystems, Foster City, CA, USA) using SYBR green (Vazyme Biotechnology, Nanjing, China) in a 10 μL reaction mixture containing 1 μL diluted cDNA, 10 μM of each primer 0.4 μL, ddH_2_O 3.2 μL, and 5 μL 2 × AceQ Universal SYBR qPCR Master Mix. The PCR reaction protocol was 95 °C for 2 min, 40 cycles of 95 °C for 15 s, and 60 °C for 30 s. The qRT-PCR results were calculated using the 2^−ΔΔCT^ method. Three biological replicates were established for each sample. The CAC and Ubi genes were used as housekeeping genes in *Prunus salicina* and pepper, respectively [2]. All the primers used in the PCR analysis are listed in (Table S1).

### 4.14. Statistical Analysis

The experimental results were organized, the corresponding graphs were plotted using Office Excel 2016, and the data were analyzed by ANOVA (*p* < 0.05) using SPSS 19.0.

## 5. Conclusions

In this study, we investigated plum fruit development across six distinct stages. Our analysis of Differentially Expressed Genes (DEGs) uncovered key genes, including *SUT* and *SWEET*, which are likely linked to sugar metabolism in plum fruits. Notably, our gene-sugar association analysis pinpointed two significant *SWEET* family genes, *PsSWEET9* and *PsSWEET2*, shedding light on sugar transport mechanisms and providing a foundation for future investigations into sugar transport in *Prunus salicina* ‘Huangguan’. Furthermore, we delved into the intriguing aspect of plum fruit coloration, particularly its shift to yellow. Transcriptomic KEGG-enrichment analysis unveiled the continued importance of the flavonoid biosynthesis pathway, consistent with findings in other plum varieties, in determining plum color. Our functional validation of an essential *PsCCS* gene in chili peppers suggests its potential role as a positive regulator in the color transformation of *Prunus salicina* ‘Huangguan’. In summary, our study highlights three pivotal anabolic pathways during plum fruit development: photosynthesis, sugar transport mechanisms, and carotenoid biosynthesis. These findings deepen our understanding of the molecular processes underpinning the development and coloration of plum fruits.

## Data Availability

The data is contained within the manuscript and Supplementary Materials.

## Supplementary Materials

The following supporting information can be downloaded at https://www.mdpi.com/article/10.3390/plants12193513/s1, Figure S1. The soluble sugars concentrations in plum during fruit development stages; Figure S2. Top 20 enriched KEGG pathways of DEGs at different developmental stages; Figure S3. Quantitative validation of DEGs associated with sugar metabolism; Figure S4. Expression analysis of the SWEET gene in ethylene-treated fruits; Figure S5. Analysis of promoter cis-acting elements and promoter transcription binding sites of the *PsSWEET* gene family; Figure S6. Hierarchical clustering of DEGs in G and VA, VB, M, P stage of plum, respectively; Figure S7. RT-qPCR analysis of the level of expression of capsanthin/capsorubin synthase in fruits at different stages of growth and development; Figure S8. Phenotypic observations of transient overexpression of the *PsCCS* gene in chili peppers; Table S1. List of gene-specific primers used for real-time quantitative PCR expression analysis; Table S2. Protein physico-chemical properties of all *PsSWEET* genes; evm.TU.Chr6.1365 (CCS) nucleotide sequence. Reference [2] has been cited in the Supplementary Material part.
