# Supplementary material for: Transcriptome Insights into Candidate Genes of the SWEET Family and Carotenoid Biosynthesis during Fruit Growth and Development in Prunus salicina ‘Huangguan’
Source: Plants (Basel). 2023 Oct 9;12(19):3513. doi: 10.3390/plants12193513 (PMC10574959; doi:10.3390/plants12193513)
Supplement: Supplementary file 1 [file plants-12-03513-s001.zip › plants-2605708-supplementary.pdf]

Supplementary Materials

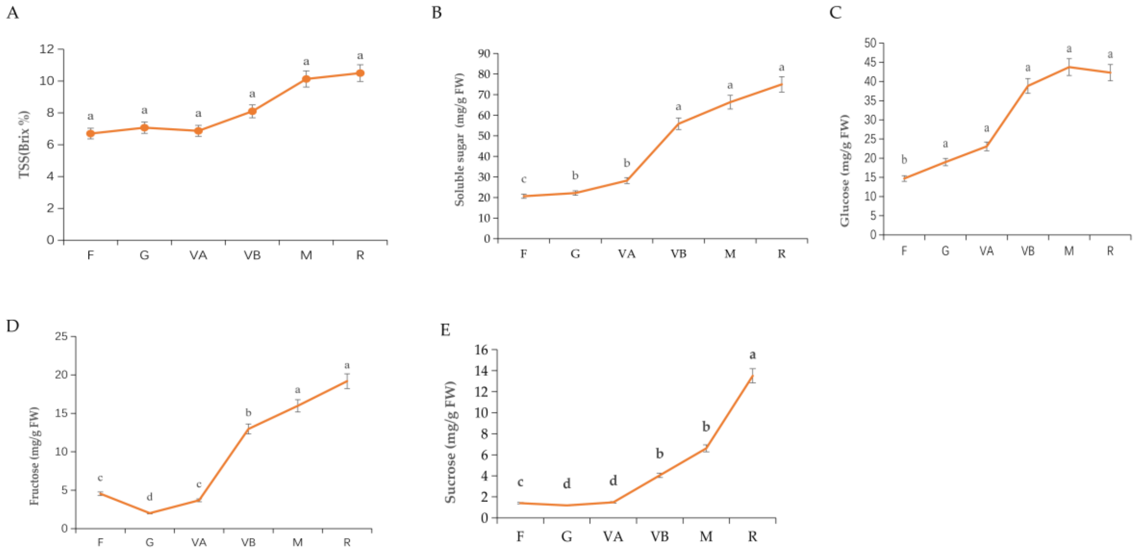

Figure S1. The soluble sugars concentrations in plum during fruit development stages. (**A**) Concentrations of TSS during plum fruit development, (**B**) Concentrations of soluble sugar during plum fruit development, (**C**) Concentrations of glucose during plum fruit development, (**D**) Concentrations of fructose during plum fruit development, and (**E**) Concentrations of sucrose during plum fruit development. All statistical differences displayed by letter were determined by ANOVA and Tukey test ($P < 0.05$).

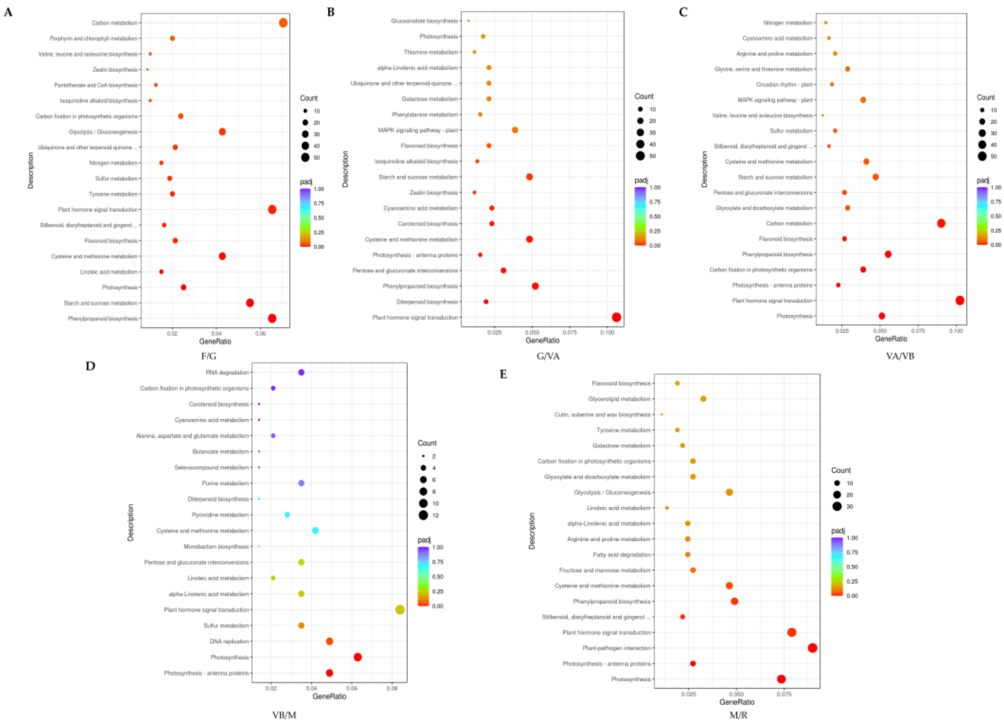

Figure S2. Top 20 enriched KEGG pathways of DEGs at different developmental stages. (**A**) Scatterplot of enriched KEGG pathway between F and G stage, (**B**) Scatterplot of enriched KEGG pathway

between G and VA stage, (**C**) Scatterplot of enriched KEGG pathway between VA and VB stage, (**D**) Scatterplot of enriched KEGG pathway between VB and M stage, (**E**) Scatterplot of enriched KEGG pathway between M and R stage.

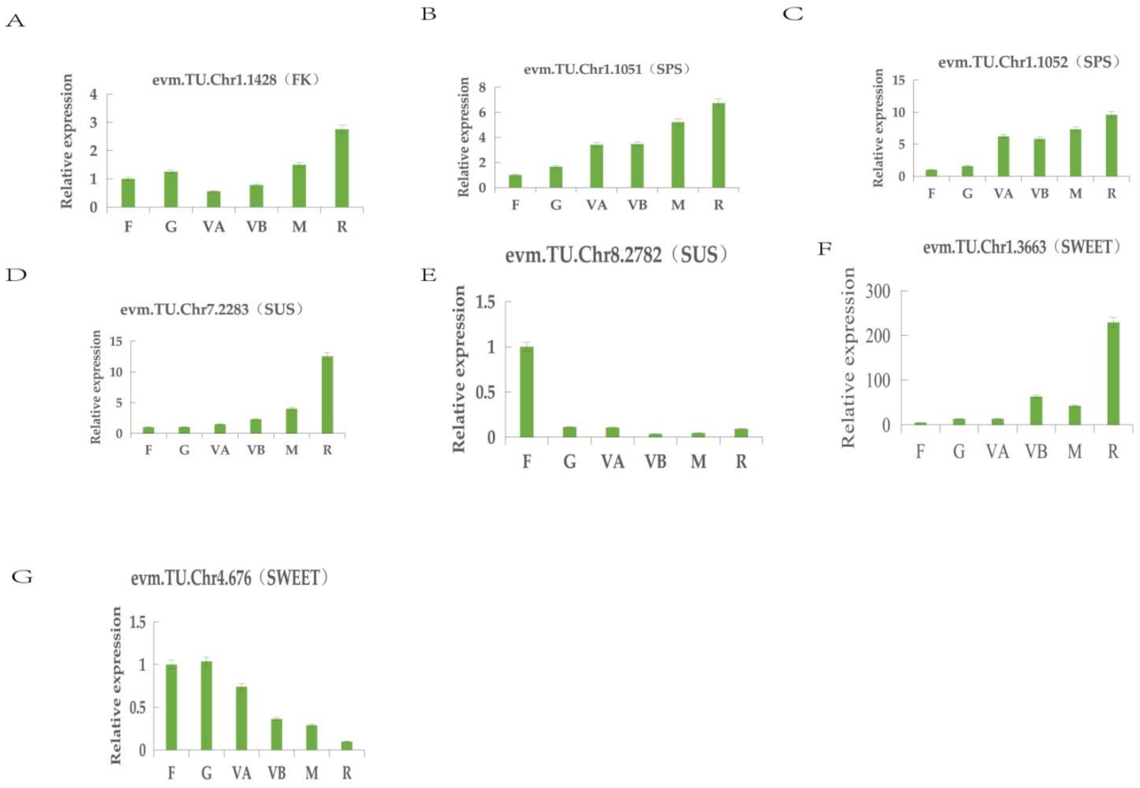

Figure S3. Quantitative validation of DEGs associated with sugar metabolism. (**A**) evm.TU.Chr1.1428 (*FK*) gene expression in different fruit development stage, (**B**) evm.TU.Chr1.1051 (*SPS*) gene expression in different fruit development stage, (**C**) evm.TU.Chr1.1052 (*SPS*) gene expression in different fruit development stage, (**D**) evm.TU.Chr7.2283 (*SUS*) gene expression in different fruit development stage, (**E**) evm.TU.Chr7.2282 (*SUS*) gene expression in different fruit development stage, (**F**) evm.TU.Chr4.676 (*SWEET*) gene expression in different fruit development stage, (**G**) evm.TU.Chr1.3363 (*SWEET*) gene expression in different fruit development stage.

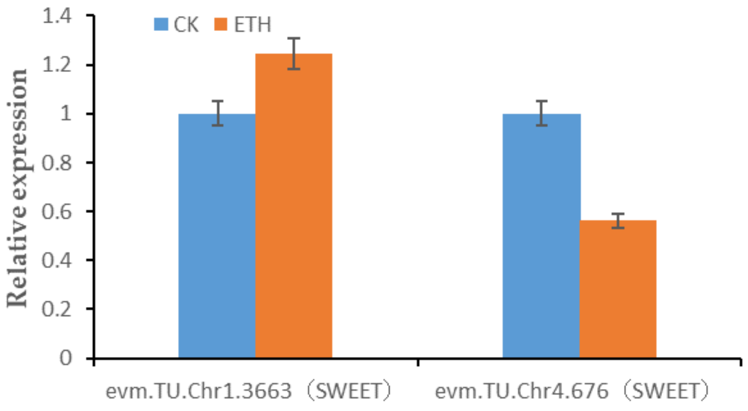

Figure S4. Expression analysis of the *SWEET* gene in ethylene-treated fruits.

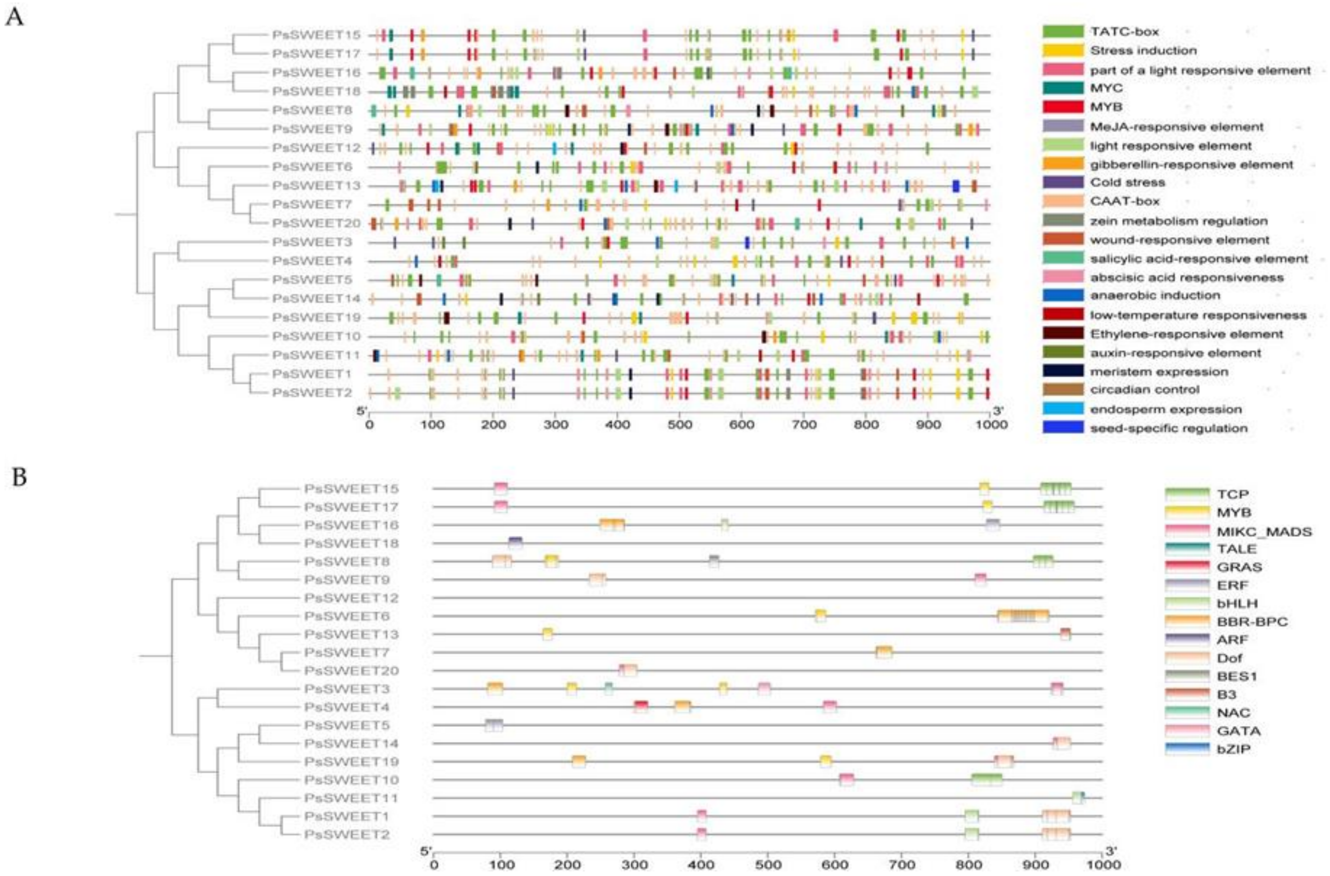

Figure S5. Analysis of promoter cis-acting elements and promoter transcription binding sites of the *PsSWEET* gene family. (**A**) cis-acting elements analysis of PsSWEET promoters, (**B**) promoter transcription binding sites analysis of *PsSWEET* gene family.

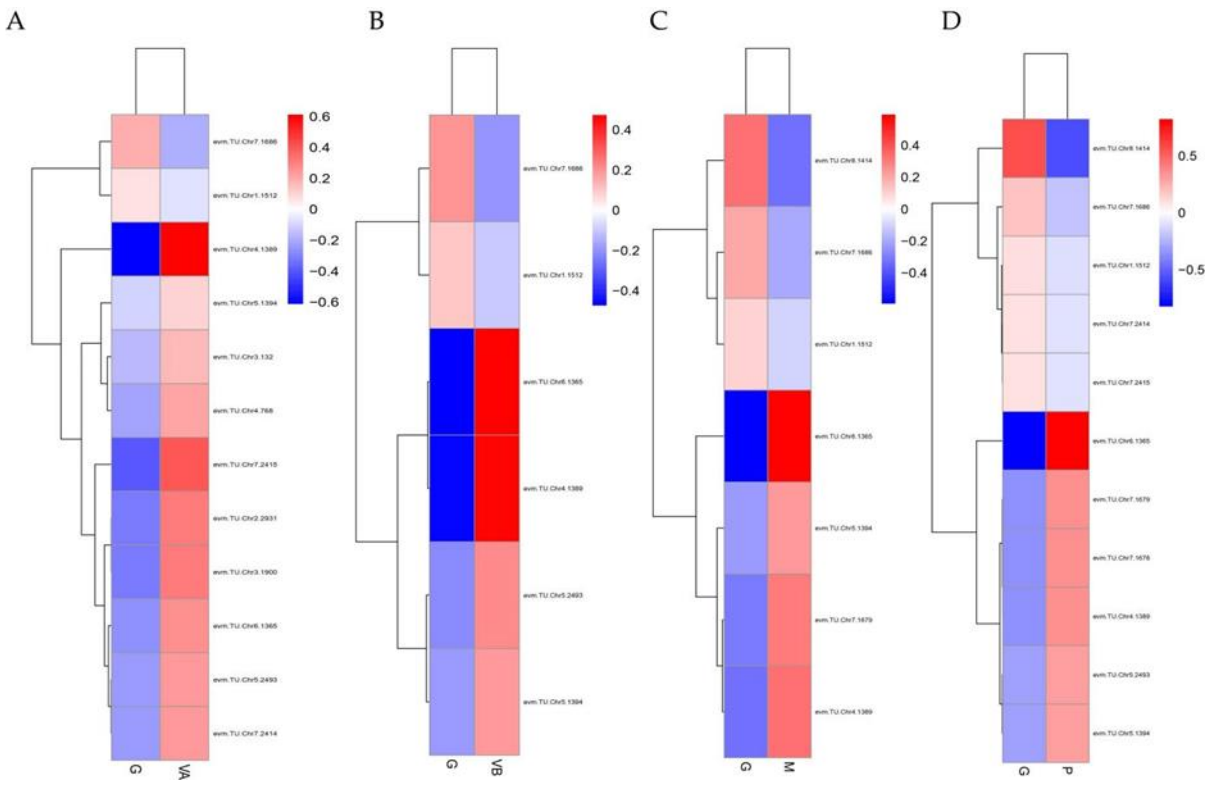

Figure S6. Hierarchical clustering of DEGs in G and VA, VB, M, P stage of plum, respectively. (**A**) DEGs between G and VA stage, (**B**) DEGs between G and VB stage, (**C**) DEGs between G and M stage,(**D**) DEGs between G and P stage. evm.TU.Chr2.2931(*bHY*), beta-carotene hydroxylase; evm.TU.Chr3.132/evm.TU.Chr3.1900 (*PSY*), phytoene synthase-like; evm.TU.Chr4.768/evm.TU.Chr4.1389 (*NCED2*/*NCED1*), 9-cis-epoxycarotenoid dioxygenase; evm.TU.Chr6.1365 (*CCS*), capsanthin/capsorubin synthase; evm.TU.C-hr3.1900 (*PSY*),phytoene synthase; evm.TU.Chr5.2493/evm.TU.Chr7.1686/evm.TU.Chr1.1512 (*CYP707A1*/*CYP707A2*/*CYP707A1*), avium abscisic acid 8'-hydroxylase 1-like; evm.TU.Chr7.2415/evm.TU.Chr7.2414 (*LCY*), lycopene epsilon cyclase; evm.TU.Chr5.1394 (*BHYD*),beta-carotene hydroxylases.

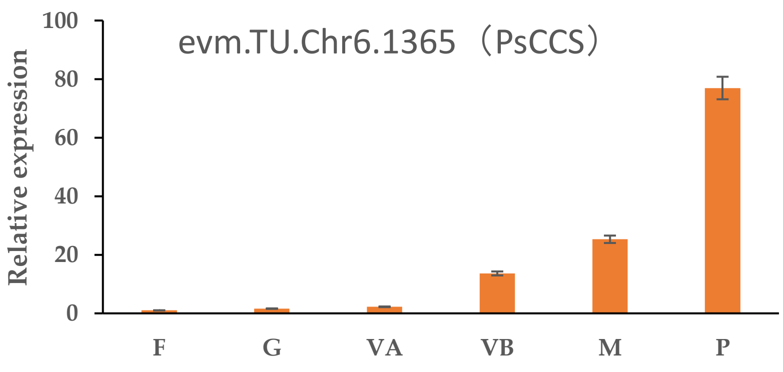

Figure S7. qRT-PCR analysis of the level of expression of capsanthin / capsorubin synthase in fruits at different stages of growth and development.

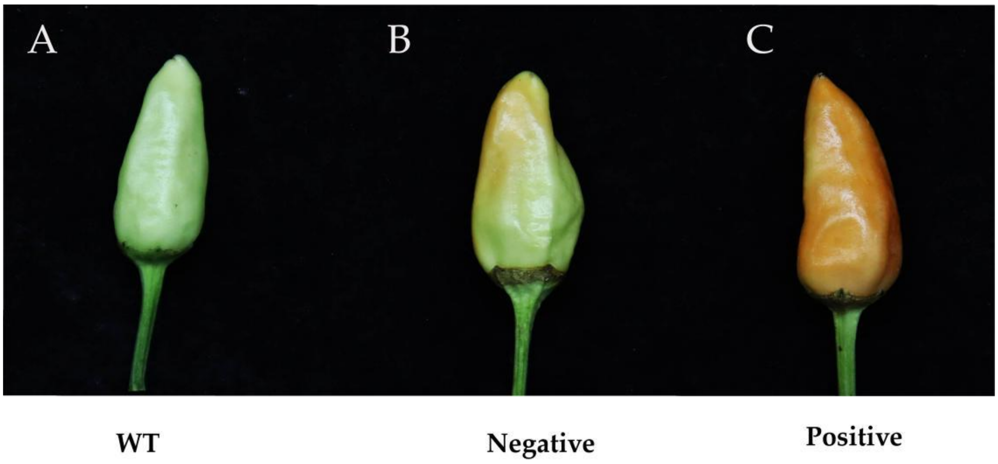

Figure S8. Phenotypic observations of transient overexpression of the *PsCCS* gene in chilli peppers. (**A**) Control of wild type, (**B**) Injection of empty plasmid strains as negative, (**C**) Injection of strains containing the *PsCCS* gene as positive.

Table S1. List of gene-specific primers used for real-time quantitative PCR expression analysis.

	Name	Forward primer (5' to 3')	Reverse primer (5' to 3')	Source
qRT-PCR	**evm.TU.Chr1.1428 (*FK*)**	GATTTTGGTTATGTGCTC	TTTACAACTGAATAA-GCC	This study
	**evm.TU.Chr1.1051 (*SPS*)**	CCTTTCAGCTCATAGTGAT	CTAGCCCAATTCTCCATC	This study
	**evm.TU.Chr1.1052 (*SPS*)**	GGTGGATTTAGAT-TACCG	TCATTGAAAGAA-GCAACC	This study
	**evm.TU.Chr7.2283 (*SUS*)**	TAAGCGTATTAA-GCAACAGG	AACACGAAGAA-TATGTG	This study
	**evm.TU.Chr8.2782 (*SUS*)**	CTTATAGCCATGAA-TAATGC	TTCCATGAACAACTCGGTA	This study
	**evm.TU.Chr1.3663 (*SWEET*)**	TTCGGAACTGTAA-TAGAAACC	CCACGTTCATAAAAC-CAA	This study
	**evm.TU.Chr4.676 (*SWEET*)**	CAATTCTCTG-GATTGCCTT	GCCACTAGTATAATAC-CAGTC	This study
	CAC	GGGATACGCTACAA-GAAGAATGAG	CTTACACTCTGGCAT-ACCACTCAA	[2]
	**evm.TU.Chr6.1365 (CCS)**	AGCAAGTGTCACAG-CATGGCATT	GCAGGCTCTCAAACTCATCAACCC	This study
	**Ubi gene (GeneBank no:AY486137.1)**	GAGCAGTGGAGTCCAG-CATTAACC	TTCTGAGTCCAGCTAC-GAGCAGTG	This study
**Gene clon-ing**	**evm.TU.Chr6.1365 (CCS)**	TCTGCAACATCTA-GAATGGG-TACTCTTCTGAGGCTATT	TGTCAC-TTCGAGCTCTTAGATA-TAATATTGTTTTGCTTC	This study

Table S2. Protein physico-chemical properties of all PsSWEET genes.

Gene name	Gene ID	Number of amino acids	Molecular weight （kD）	PI	Instability index	Aliphatic index	GRAVY	Signal peptide	Number ofTransmembrane domains	Subcellular localization
PsSWEET1	evm.TU.Chr1.3629	282	31.38	8.34	35.97	113.69	0.66	YES	7	Cell membrane
PsSWEET2	evm.TU.Chr1.3663	282	31.38	8.34	35.97	113.69	0.66	YES	7	Cell membrane
PsSWEET3	evm.TU.Chr1.4665	252	27.77	7.77	41.62	116.87	0.712	YES	7	Cell membrane
PsSWEET4	evm.TU.Chr2.1311	243	26.85	9.16	41.07	127.2	0.804	YES	7	Cell membrane
PsSWEET5	evm.TU.Chr2.2409	147	16.58	9.6	35.84	126.6	0.758	YES	4	Chloroplast
PsSWEET6	evm.TU.Chr3.2937	226	24.85	9.07	33.35	106.15	0.478	NO	5	Cell membrane
PsSWEET7	evm.TU.Chr3.333	133	15.21	8.69	38.16	130.3	0.807	YES	4	Cell membrane
PsSWEET8	evm.TU.Chr4.1434	235	26.43	8.63	43.66	120.72	0.815	NO	7	Cell membrane
PsSWEET9	evm.TU.Chr4.676	235	25.95	9	36.78	121.91	0.91	NO	7	Cell membrane
PsSWEET10	evm.TU.Chr5.1013	293	33.08	5.97	41.93	126.38	0.742	YES	7	Cell membrane
PsSWEET11	evm.TU.Chr5.1014	290	32.94	8.06	42.67	110.86	0.516	NO	6	Cell membrane
PsSWEET12	evm.TU.Chr5.1208	253	28.02	9.3	38.04	134.07	0.841	NO	7	Cell membrane
PsSWEET13	evm.TU.Chr5.728	182	20.77	9.23	60.98	133.74	0.903	NO	5	Cell membrane
PsSWEET14	evm.TU.Chr6.3661	273	30.89	9.55	39.73	104.98	0.514	YES	7	Cell membrane
PsSWEET15	evm.TU.Chr8.154	288	31.34	9.65	41.32	113.44	0.793	YES	8	Cell membrane
PsSWEET16	evm.TU.Chr8.155	141	15.82	9.83	36.48	123.83	0.469	NO	3	Cell membrane
PsSWEET17	evm.TU.Chr8.220	288	31.34	9.65	41.32	113.44	0.791	YES	8	Cell membrane
PsSWEET18	evm.TU.Chr8.221	105	11.90	9.74	44.56	122.67	0.25	NO	2	Cell membrane
PsSWEET19	evm.TU.Chr8.2618	284	31.60	8.57	39.81	111.27	0.693	YES	6	Cell membrane
PsSWEET20	evm.TU.Chr8.961	106	11.96	5.51	35.2	147.92	0.885	YES	3	Chloroplast

evm.TU.Chr6.1365 (CCS) nucleotide sequence: 1503bp
ATGGGTACTCTTCTGAGGCTATTTTCACCACCACCACCAGCAACCCAGAG-
CAGCCAATTTTTTCATTCATTCAATCCTCCATTTTCATTTCCAAGAACCTGTGGCACATCTTCGAGAAATACTCCTTCCAAAGCACAGAGCAGCGAATGTG
GAAATTTTCTTGATTTAAAACCGGTGGCAAAACCTGAATTCTTAGATTTCAACAT-
ACCCCGGTTTGACACATCCACTAGGTCTTCCTTAGACGTGATCATCATTGGCACTGGCCCTGCCGGGCTTCGCCTTGCAGAGCAAGTGTCACAGCATGGC
ATTAAGGTATGTTGTGTTGATCCTTCACCGCAATCTTTGTGGCCAAATACCTATTCTGTTT-
GGGTTGATGAGTTTGAGAGCCTGCAGCTTGAGAGTTGCTTAGAAAAAACATGGCCTAGGTCTTGCTTTTATATTGATGAAGACAATGTCAAGTATATAGA
CCGCCCTTTCGGTCGAATTAGCAGGAAGAAATTCAAGACTTTGCTAATGGA-
GAGGTGCCTCTCTAATGATGTCAAGTTTCATAAGGCCAAGGTTTTGAAAATTGAGCACCAAGAGTTTGAATCTTCGATATTCTGTGACGATGGCAATGAG
CTCAAGGCAAGCCTAATTGTTGATGCTAGTGGGTTTGCAAGCACTTTCACGGAG-
TATGACAAACCAAGGAACCATGGCTATCAGACTGGCCATGGCATTTTGGCTGAAGTGGATTTTCACCCTTTTGACTTGGATAAGGTTGTTCAGTTGGATTG
GAGAGACTCCCATCTCAGAAATGAGCCTTCAATGCTCACTACAAATTCAAGAT-
TTCCAACTTTTCTCTTTGCCATGCCATTTGATTCAAGCCTAATATATTTAATGGAAACTTCATTGGTTAGTAGGCCAGTGCTGCCTTATGAGGAGGTGAAG
AAGAGAATGGTTGCAAGGCTAAGGCATTTGGGAATTAGAGTGAAGAGGGTTTTGGAGGCTGA-
GAAGTGTTTGATCCCAATGGGAGGTCCTCTTCCTCGGATGTCTCAAGAAGTGATGGCTATGGGAGGAACTTCCGGGCTTGTCAATCCTTCAGCCGGGTAC
ATGGTGGCGTCAGCCATGGCTCTAGCCCCGGTGCTAGCCGAGGCCATTGTGCAGTGCCTT-
GGCTCGACCCCAATGATCCGAGGGCAGCCACTTTATGATAGAGTGTGGAAGGGGCTGTGGCCAATTGAGAGGAGATGTGTTAGGGAGTTTTACTCTTTTG
GGATGGAGACTTTGTTGACGCTTGATCTGAATGAGACTCGAAGGTTCTTTGATGCTTTCTTT-
GACTTGGATCCGTATTACTTGCACGGCTATCTTTCATCAAGGTTGTCCCCAACAGAGCTTGCCCTTTTCAGTTTATCTATGTTTGGCAATGCCTCTGTTTCAT
CTAGAGTTGAACTTCTTACAAAGTCTGCTGTACCCTTCGTTAAAATGATAGGTAATCTAGCACTTGAAGCAAAACAATATTATATCTAA
